# Exercise-based Rehabilitation in Cardio-renal-pulmonary Multimorbidity: Shared Pathophysiology, Functional Phenotypes, and Clinical Implications

**DOI:** 10.1298/ptr.R0044

**Published:** 2026-07-07

**Authors:** Toshimi SATO, Yuichiro SASAMOTO, Daisuke SUZUKI, Shun MONOE, Keita KANEKO, Shinichiro MORISHITA

**Affiliations:** 1 Department of Physical Therapy, Graduate School of Health Sciences, Fukushima Medical University, Japan; 2 Department of Physical Therapy, School of Health Sciences, Fukushima Medical University, Japan; 3 Department of Rehabilitation, Ohta General Hospital Foundation, Ohta Nishinouchi Hospital, Japan; 4 Department of Rehabilitation, Southern Tohoku General Hospital, Japan; 5 Department of Rehabilitation, Wakamatsu Aoi Clinic, Japan; 6 Department of Rehabilitation, Hoshi General Hospital, Japan

**Keywords:** Heart failure, Chronic kidney disease, Chronic obstructive pulmonary disease, Multimorbidity, Rehabilitation

## Abstract

Heart failure (HF), chronic kidney disease (CKD), and chronic obstructive pulmonary disease (COPD) frequently coexist and collectively constitute a high-risk multimorbid state characterized by shared pathophysiology, compounded exercise intolerance, frailty, and worse clinical outcomes. These overlapping conditions interact through several mechanisms, including neurohormonal activation, systemic inflammation, oxidative stress, endothelial dysfunction, hemodynamic interactions, and skeletal muscle abnormalities. These mechanisms restrict functional capacity through both central and peripheral pathways. Exercise-based rehabilitation has emerged as an essential nonpharmacological intervention across cardiovascular, renal, and pulmonary medicine and is supported by robust evidence for improvements in exercise capacity and health-related quality of life in single-organ disease populations. However, patients with overlapping cardio-renal-pulmonary impairment remain underrepresented in rehabilitation trials, and evidence for the triple overlap of HF, CKD, and COPD remains particularly scarce. This narrative review synthesizes current evidence on the pathophysiology of cardio-renal-pulmonary multimorbidity and the mechanisms of exercise intolerance. Furthermore, it summarizes functional phenotypes, clinical assessments, and the effects of exercise-based rehabilitation across single-organ and overlapping disease states. Finally, practical implications for individualized exercise prescription, delivery models, and safety monitoring are discussed. We propose a phenotype-guided, function-centered framework for rehabilitation in this complex population and outline key research priorities to strengthen the evidence base for this high-risk, underserved population.

## Introduction

The global burden of chronic noncommunicable diseases is increasingly influenced by multimorbidity, commonly defined as the coexistence of 2 or more chronic conditions in the same individual. Among the most clinically consequential combinations is the overlap of cardiac, renal, and pulmonary diseases, which is not only additive comorbidity but also a unique multimorbid state characterized by shared pathophysiology, compounded exercise intolerance, accelerated functional decline, and excess mortality. Heart failure (HF) affects more than 64 million individuals worldwide and is commonly complicated by chronic kidney disease (CKD), which is present in several patients and is consistently linked to worse symptoms, more frequent hospitalization, and poorer survival^1,2)^. Chronic obstructive pulmonary disease (COPD) is also highly prevalent in adults and commonly coexists with HF due to shared risk factors, including aging, smoking, physical inactivity, and systemic inflammation^3)^. The resulting cardio-renal-pulmonary multimorbidity imposes a burden that exceeds that of any single disease alone and includes recurrent decompensation, progressive disability, and high healthcare utilization^4)^.

Exercise intolerance is a cardinal and often disabling manifestation of multimorbidity. In affected patients, reduced cardiac reserve, ventilatory limitation, impaired peripheral oxygen extraction, autonomic dysfunction, anemia, skeletal muscle abnormalities, and frailty converge to limit physical performance and daily function^5–7)^.

Exercise-based rehabilitation—including cardiac rehabilitation (CR), renal rehabilitation, and pulmonary rehabilitation (PR)—is an established component of care in cardiovascular and pulmonary diseases and is an increasingly recognized intervention for CKD, with evidence for improvements in exercise capacity, physical function, symptoms, and health-related quality of life (QOL) across these populations. Contemporary HF guidance supports supervised exercise training as an essential element of comprehensive care, and a joint scientific statement from the American Heart Association and American College of Cardiology concluded that supervised exercise training in chronic, stable HF is safe and clinically beneficial^8–11)^. Renal rehabilitation has similarly been described as a feasible and promising multidimensional strategy in CKD, particularly for improving physical function and QOL^12)^. However, patients with multimorbidity remain systematically underrepresented in rehabilitation trials and are frequently underserved in clinical practice, partly because of uncertainty regarding feasibility, safety, and expected benefit when multiple organ systems are simultaneously impaired.

This narrative review addresses this gap by: (1) describing the shared pathophysiology of cardio-renal-pulmonary multimorbidity; (2) examining cardiopulmonary exercise testing (CPET)-derived functional phenotypes and mechanisms of exercise intolerance across HF, CKD, and COPD and their overlap; (3) synthesizing available evidence for exercise-based rehabilitation in single-organ and overlapping disease states; and (4) proposing clinical implications and future priorities for individualized rehabilitation in this high-risk, functionally vulnerable population. Consistent with the objectives of a narrative review, we prioritized clinical practice guidelines, randomized controlled trials (RCTs), meta-analyses, and mechanistic studies most relevant to exercise intolerance, functional phenotypes, rehabilitation delivery, and safety across HF, CKD, COPD, and their overlap (Fig. 1).

**Fig. 1.**
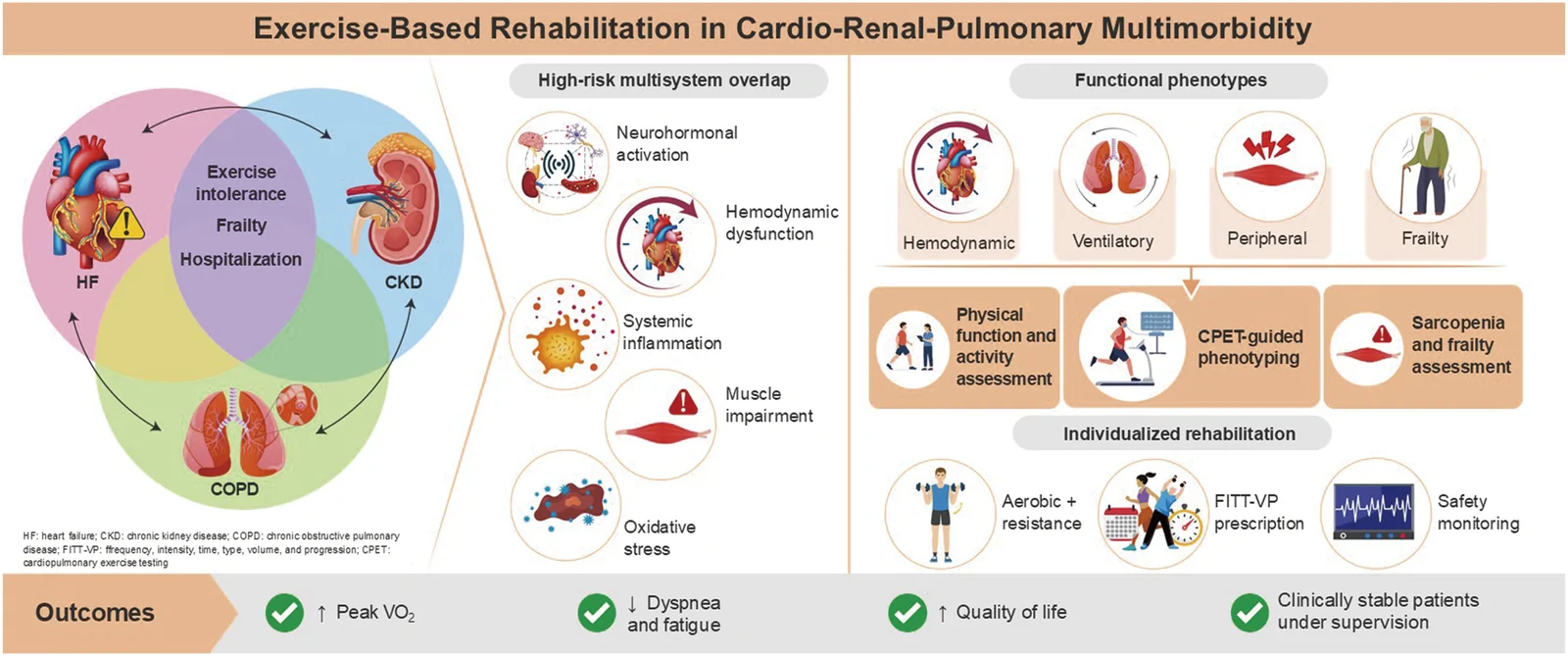
Conceptual framework of cardio-renal-pulmonary multimorbidity and phenotype-guided, exercise-based rehabilitation. HF, CKD, and COPD interact through shared and bidirectional mechanisms, including neurohormonal activation, systemic inflammation, oxidative stress, endothelial dysfunction, hemodynamic interdependence and congestion, hypoxia, uremic toxicity, and skeletal muscle dysfunction. These processes converge to produce exercise intolerance through central hemodynamic limitation, ventilatory inefficiency, impaired peripheral oxygen delivery and utilization, anemia, autonomic dysfunction, sarcopenia, and frailty, resulting in progressive functional decline and adverse clinical outcomes. The figure highlights a function-centered rehabilitation framework in which cardiopulmonary exercise testing and multidomain assessment guide phenotype-specific exercise prescription, including aerobic training, resistance training, selected inspiratory muscle training, individualized progression, and safety monitoring. This integrated approach applies cardiac, renal, and PR principles to target exercise capacity, physical function, symptoms, activities of daily living, and health-related QOL in patients with cardio-enal-pulmonary multimorbidity. HF, heart failure; CKD, chronic kidney disease; COPD, chronic obstructive pulmonary disease; FITT-VP, frequency, intensity, time, type, volume, and progression; CPET, cardiopulmonary exercise testing; peak VO_2_, peak oxygen uptake; PR, pulmonary rehabilitation; QOL, quality of life

## Pathophysiology of Cardio-renal-pulmonary Multimorbidity

Neurohormonal dysregulation is a core shared pathophysiological mechanism across HF, CKD, and COPD. In HF, reduced cardiac output activates the renin–angiotensin–aldosterone system (RAAS), the sympathetic nervous system (SNS), and arginine vasopressin, resulting in sodium and water retention, vasoconstriction, and maladaptive cardiac remodeling^2)^. These responses initially preserve perfusion but ultimately perpetuate a cycle of congestion, end-organ injury, and progressive dysfunction. CKD intensifies these responses through impaired renal autoregulation, reduced natriuretic peptide clearance, and intrinsic RAAS hyperactivation, thereby further increasing afterload and promoting myocardial and renal fibrosis^13)^. COPD contributes through hypoxia-driven SNS activation, increased myocardial oxygen demand, altered volume regulation, and skeletal muscle catabolism, thereby reinforcing the neurohormonal environment underlying multiorgan dysfunction^3,14)^.

Systemic inflammation and oxidative stress form an additional shared substrate across all 3 conditions and are magnified in multimorbidity. Elevated levels of tumor necrosis factor-α, interleukin-6, C-reactive protein, galectin-3, and oxidative stress mediators have been described in HF, CKD, and COPD, and their coexistence generates a pro-inflammatory state that accelerates disease progression^15,16)^. In HF with preserved ejection fraction (HFpEF), inflammatory signaling related to obesity, hypertension, and diabetes mellitus contributes to coronary microvascular dysfunction, myocardial fibrosis, and diastolic dysfunction^16)^. In CKD, uremic toxins and advanced glycation end-products impair endothelial function and promote vascular oxidative stress, whereas COPD-related inflammation increases cardiovascular risk and skeletal muscle catabolism through proteolytic pathways^3,17,18)^. These intersecting pathways contribute to skeletal muscle wasting, endothelial dysfunction, and progressive multiorgan injury that together underlie frailty and severe exercise limitation in patients with multimorbidity.

Hemodynamic interdependence is another defining feature of cardio-renal-pulmonary multimorbidity. In HF, elevated left-sided filling pressures are transmitted to the pulmonary venous circulation, causing pulmonary hypertension, right ventricular dysfunction, and renal venous congestion^2)^. Increased central venous pressure directly impairs glomerular filtration and tubular function and is an essential mechanism underlying cardiorenal syndrome^14)^. Pulmonary hypertension, common in both HF and COPD, further increases right ventricular afterload and may precipitate right-sided failure, worsening renal perfusion and systemic congestion^4)^. In COPD, dynamic hyperinflation can decrease venous return and blunt right ventricular preload, thereby restricting the capacity to increase cardiac output during exercise^7,19)^. The resulting instability in preload reserve, cardiac output, and ventilation–perfusion matching has major implications for rehabilitation prescription and monitoring.

The cardiac, renal, and pulmonary systems also engage in bidirectional crosstalk mediated by shared neurohormonal, inflammatory, and hemodynamic pathways. Renal dysfunction in HF is not only a passive consequence of low perfusion but also an active contributing factor to disease progression through fluid retention, neurohormonal activation, and uremic cardiomyopathy^13,17)^. In contrast, cardiac dysfunction in CKD is influenced by volume overload, hypertension, anemia, and uremic toxins that promote left ventricular hypertrophy, diastolic dysfunction, and accelerated atherosclerosis^1,17)^. Pulmonary disease interacts with both systems through hypoxia, systemic inflammation, and vascular dysfunction, while studies have identified shared molecular pathways—including nuclear factor kappa B (NF-κB) signaling, transforming growth factor beta (TGF-β)-mediated fibrosis, and mitochondrial dysfunction—that may serve as future therapeutic targets across the cardio-renal-pulmonary axis^4,18)^.

## Functional Phenotypes and Exercise Intolerance

Exercise intolerance in HF is multifactorial and involves both central hemodynamic limitation and peripheral skeletal muscle abnormalities, with the relative contribution varying according to HF phenotype. CPET classically demonstrates reduced peak oxygen uptake (peak VO_2_), an elevated minute ventilation/carbon dioxide production (VE/VCO_2_) slope, and an early anaerobic threshold^5,20)^. In heart failure with reduced ejection fraction (HFrEF), peak VO_2_ is often constrained by impaired cardiac output augmentation resulting from reduced stroke volume reserve and chronotropic incompetence. In HFpEF, peripheral factors—including impaired oxygen extraction, skeletal muscle mitochondrial dysfunction, and chronotropic incompetence—appear to play a particularly important role^5,21)^. Hyperlactatemia at submaximal exercise reflects impaired peripheral oxygen utilization and has been linked to reduced exercise capacity in HFpEF^22)^. The VE/VCO_2_ slope, a marker of ventilatory inefficiency reflecting dead-space ventilation and chemoreflex sensitivity, is elevated in both HFrEF and HFpEF^20,23)^.

Exercise intolerance in CKD appears to be influenced predominantly by peripheral mechanisms, distinguishing it from the more overt central hemodynamic limitation typical of advanced HFrEF^24)^. CPET studies have demonstrated reduced peak VO_2_ across the spectrum of CKD severity, with progressive deterioration as the estimated glomerular filtration rate (eGFR) decreases. Near-infrared spectroscopy and related assessments indicate that impaired peripheral oxygen extraction is disproportionate to central cardiac limitations^24,25)^. Potential contributors include uremic myopathy, anemia, autonomic dysfunction, physical inactivity, and chronic inflammation-mediated muscle catabolism^6,24)^. Systematic reviews and meta-analyses confirm marked reductions in cardiopulmonary reserve in CKD compared with that in matched controls^26,27)^. In patients with concomitant HF and CKD, peak VO_2_ is typically further reduced compared with that in patients with either condition alone, suggesting additive impairment of both central and peripheral oxygen transport mechanisms^27)^.

In COPD, exercise intolerance is frequently dominated by ventilatory limitation, including dynamic hyperinflation, impaired gas exchange, and increased dead-space ventilation. However, skeletal muscle dysfunction and cardiovascular deconditioning also contribute substantially, particularly in advanced disease and in the presence of cardiovascular comorbidity. CPET in COPD may demonstrate reduced peak VO_2_, an elevated VE/VCO_2_ slope, early ventilatory limitation, and an impaired oxygen pulse^7,28,29)^. When COPD coexists with HF, exercise capacity is consistently lower than in either condition alone, reflecting additive ventilatory and hemodynamic limitations^7,30–32)^. In the Fitness Registry and the Importance of Exercise National Database registry, concomitant HF and COPD were associated with the lowest peak VO_2_, lower end-tidal partial pressure of carbon dioxide (PETCO_2_) and greater ventilatory inefficiency than HF or COPD alone^29)^. These findings support the concept that overlap syndromes should be assessed using integrated functional phenotyping rather than disease labels alone.

Sarcopenia, frailty, and disability represent clinically important downstream functional phenotypes across HF, CKD, and COPD. Sarcopenia is highly prevalent across all 3 conditions and becomes more common with multimorbidity^33)^. Shared mechanisms include chronic inflammation, anorexia and malnutrition, physical inactivity, uremic toxin-mediated muscle catabolism, corticosteroid exposure, and endocrine dysregulation^6,15)^. Frailty—whether defined by the Fried phenotype or by clinical frailty scales—is likewise common and strongly linked to hospitalization, mortality, and rehabilitation noncompletion^34–36)^. The resulting disability in activities of daily living contributes to poor health-related QOL, caregiver burden, and institutionalization. In patients with multimorbidity, the combined phenotype of low exercise capacity, sarcopenia, frailty, and disability has direct implications for exercise prescription, monitoring, and goal setting.

## Effects of Exercise-based Rehabilitation in Single-organ Disease

The evidence base for exercise-based CR in HF is substantial and has expanded with contemporary RCTs and meta-analyses. In HFrEF, CR consistently improves peak VO₂ by clinically meaningful margins, reduces ventilatory inefficiency, and improves health-related QOL as evaluated using disease-specific instruments^8,37)^. High-intensity interval training may yield larger gains in peak VO_2_ than moderate continuous training in selected HFrEF populations, although both approaches improve symptoms and functional capacity^37)^. In the Heart Failure: A Controlled Trial Investigating Outcomes of Exercise Training (HF-ACTION), exercise-based training improved exercise capacity and QOL. Although the unadjusted primary composite endpoint of all-cause mortality or hospitalization was not significantly reduced, adjusted analyses demonstrated modest reductions in clinical events, supporting the incorporation of structured exercise training into guideline-based HF care^9)^.

In HFpEF, where pharmacological strategies have historically shown limited benefit for symptoms and functional capacity, exercise training has emerged as a particularly essential intervention. Multiple meta-analyses confirm that supervised exercise training improves peak VO_2_ and health-related QOL in HFpEF, with combined endurance and resistance training showing particular promise^10,11,37–39)^. A recent multicenter RCT of combined endurance and resistance training in HFpEF did not meet its primary composite endpoint; however, clinically relevant improvements in peak VO_2_ and New York Heart Association functional class were observed; however, changes in diastolic function parameters were not significantly different between groups^40)^. Taken together, current evidence supports exercise training as a clinically beneficial and safe component of care in chronic, stable HFpEF^9)^.

Exercise-based rehabilitation in CKD and dialysis populations is supported by a growing body of RCTs and meta-analyses showing consistent benefits for physical function, exercise capacity, and QOL. Intradialytic exercise is feasible and generally safe and has been linked to improvements in physical performance and patient-reported outcomes^41–43)^. A training program combining aerobic exercise and strength training may be an effective intervention for improving functional outcomes in patients undergoing hemodialysis^44,45)^. Mechanistic studies in animal models indicate that exercise favorably affects oxidative stress, nitric oxide signaling, glomerulosclerosis, and tubular injury^46–49)^. However, although exercise appears safe in terms of renal parameters and may improve vascular and cardiopulmonary function, evidence for delayed CKD progression, reduced proteinuria, or improved eGFR trajectory remains heterogeneous and insufficient for definitive conclusions regarding renal endpoints^12,50,51)^.

PR remains a cornerstone nonpharmacological intervention for COPD, with consistent benefits across disease severity for exercise capacity, dyspnea, and health-related QOL^52–54)^. Endurance and resistance training are complementary core components of PR, and selected patients may also benefit from high-intensity interval training or inspiratory muscle training^53,55,56)^. Contemporary guidance strongly supports PR in stable COPD and after hospitalization for exacerbation and provides either center-based PR or telerehabilitation according to access and patient preference^52,54)^. Importantly, PR benefits are generally preserved in patients with cardiovascular and metabolic comorbidities, including HF; however, these patients may require more individualized intensity prescriptions and closer monitoring^57,58)^.

## Exercise-based Rehabilitation in Multimorbid Populations

CKD is among the most prevalent comorbidities in HF and may attenuate—but does not eliminate—the benefits of CR. Observational studies have reported that patients with HF and CKD can achieve significant improvements in peak VO_2_, walking distance, and health-related QOL during CR, although gains may be smaller than in patients without CKD and may diminish with increasing CKD severity^59,60)^. Importantly, advanced CKD should not be considered an automatic reason to withhold CR, particularly when functional limitation is substantial and the patient is clinically stable^59,61)^. Exercise training in patients with HF and CKD appears safe in terms of renal parameters; however, evidence for modification of CKD progression or improvement in eGFR trajectory remains heterogeneous and limited^50,51,59–61)^.

HF-COPD overlap presents a distinctive rehabilitation challenge because both conditions impose different but additive constraints on exercise. Standard CR and PR protocols often necessitate adaptation to account for concurrent ventilatory and hemodynamic limitations^62,63)^. Although evidence regarding this CR–PR model is limited, it has been shown that providing elderly patients with coexisting HF and COPD with a personalized program that combines regular aerobic exercise, bodyweight resistance training, and remote education on physical activity and disease management—delivered by physical therapists and nurses—can improve functional outcomes and QOL0^64)^. Audit data further suggest that completion rates and gains are lower in overlap populations than in single-disease populations, underscoring the need for individualized approaches that address both cardiac and pulmonary constraints^65)^.

The triple overlap of HF, CKD, and COPD is the most complex and highest-risk rehabilitation scenario; however, dedicated trials are virtually absent. Epidemiological and cohort data suggest that this combination is linked to severe exercise intolerance, high frailty prevalence, recurrent hospitalization, and poor survival^4)^. Available evidence from observational studies, pilot trials, subgroup analyses, and CPET-based assessments suggests that meaningful functional gains remain achievable with adapted, supervised programs that prioritize safety, realistic goal setting, and close monitoring^66,67)^. However, the evidence base remains inadequate to define optimal modality, intensity, or delivery model in this population, underscoring a major unmet need in contemporary rehabilitation research (Table 1).

**Table 1.** Evidence summary of exercise-based rehabilitation across single-organ and overlapping cardio-renal-pulmonary disease states

Population/disease state	Dominant exercise-limiting features	Main rehabilitation evidence	Consistently improved outcomes	Key references
HFrEF	Low cardiac output reserve, chronotropic incompetence, ventilatory inefficiency	Randomized trials and meta-analyses support cardiac rehabilitation. Functional and quality-of-life benefits are consistent; HF-ACTION suggested modest event reduction after adjustment	Peak VO_2_, symptoms, quality of life, ventilatory efficiency	^8–10)^
HFpEF	Peripheral dysfunction, impaired oxygen extraction, chronotropic limitation, ventilatory inefficiency	Meta-analyses and recent trials support supervised exercise training. Functional gains are consistent; cardiac structural changes are less clear	Peak VO_2_, NYHA class, quality of life	^9,11,37–40)^
CKD/dialysis	Peripheral limitation, uremic myopathy, anemia, autonomic dysfunction	Renal rehabilitation and intradialytic exercise are feasible and generally safe, with consistent benefits in physical function and patient-reported outcomes	Physical function, exercise capacity, quality of life	^12,41–45,50,51)^
COPD	Ventilatory limitation, hyperinflation, gas exchange impairment, dyspnea, deconditioning	Pulmonary rehabilitation consistently improves symptoms and performance across disease severity; center-based and selected home/tele models are supported	Exercise capacity, dyspnea, quality of life	^52–54,56,57)^
HF + CKD overlap	Combined central and peripheral limitation; volume shifts, anemia, reduced renal reserve	Observational studies and subgroup analyses show that cardiac rehabilitation can improve exercise capacity and quality of life, although gains may be smaller in advanced CKD	Functional capacity, walking performance, quality of life	^50,51,59–61)^
HF + COPD overlap	Additive hemodynamic and ventilatory constraints	Several studies have demonstrated the effectiveness of cardiopulmonary rehabilitation that combines aerobic exercise, strength training, respiratory muscle training, and symptom-based training	Function, symptoms, quality of life	^62–65)^
HF + CKD + COPD (triple overlap)	Severe multi-system limitation with frailty and recurrent instability	Direct trials are lacking. Current support is indirect, based on cohort data, pilot studies, subgroup analyses, and CPET-based observations	Selected patients may still achieve functional gains	^4,66,67)^

HFrEF, heart failure with reduced ejection fraction; HFpEF, heart failure with preserved ejection fraction; CKD, chronic kidney disease; COPD, chronic obstructive pulmonary disease; HF, heart failure; HF-ACTION, Heart Failure: A Controlled Trial Investigating Outcomes of Exercise Training; peak VO_2_, peak oxygen uptake; NYHA, New York Heart Association; CPET, cardiopulmonary exercise testing

## Clinical Implications and Exercise Prescription

Comprehensive preparticipation assessment is essential for safe and effective exercise-based rehabilitation in cardio-renal-pulmonary multimorbidity. Whenever feasible, CPET should be employed to characterize the dominant mechanism underlying exercise limitation and guide individualized prescription^5,7)^. At minimum, evaluation should include functional capacity testing, cardiac assessment (e.g., echocardiography and natriuretic peptides), renal indices (eGFR, electrolytes, and hemoglobin), pulmonary function (spirometry and oxygen saturation), frailty screening, and nutritional assessment^9,51)^. A phenotype-guided framework for assessment, exercise prescription, and monitoring is summarized in Table 2. Risk stratification should determine absolute contraindications, such as acute decompensation, unstable arrhythmia, severe valvular disease, acute pulmonary embolism, or uncontrolled resting hypoxemia, as well as relative contraindications, including severe anemia, major electrolyte disturbance, or very recent hospitalization.

**Table 2.** Phenotype-guided assessment, exercise prescription, and safety monitoring in cardio-renal-pulmonary multimorbidity

Functional phenotype	Typical clinical/CPET profile	Key baseline assessment priorities	Exercise prescription emphasis	Monitoring priorities during rehabilitation	Representative references
Hemodynamic-limited phenotype	Low peak VO_2_, impaired cardiac output reserve, chronotropic incompetence, and possible elevation of filling pressures	Echocardiography, natriuretic peptides, rhythm assessment, blood pressure response, and functional capacity testing	Moderate-intensity aerobic training with gradual progression; interval approaches may be considered in selected stable patients; resistance training should be added as tolerated	Heart rate response, blood pressure, symptoms of congestion, arrhythmia, and exertional intolerance	^5,8,9,20)^
Ventilatory-limited phenotype	Elevated VE/VCO_2_ slope, dyspnea, early ventilatory limitation, dynamic hyperinflation, and desaturation risk	Spirometry, oxygen saturation, symptom burden, inhaler optimization, and dyspnea characterization	Aerobic training with symptom-guided intensity; interval training and inspiratory muscle training may be useful adjuncts; combine with resistance training whenever feasible	Oxygen saturation, Borg dyspnea score, recovery time, ventilatory distress, and need for supplemental oxygen	^7,28,29,52,56,62)^
Peripheral-limited phenotype	Reduced peak VO_2_ with disproportionate impairment of peripheral oxygen extraction/utilization, deconditioning, and muscle weakness	Muscle strength/function testing, hemoglobin, renal indices, nutritional assessment, and physical activity history	Combined aerobic plus resistance training with progressive loading and emphasis on restoring muscle function and exercise tolerance	Perceived exertion, fatigue, post-exercise recovery, and renal/hematologic status where relevant	^24–26,44,51)^
Frailty/sarcopenia-dominant phenotype	Low gait speed, weakness, disability, low physiological reserve, and poor tolerance of conventional protocols	Frailty screening, sarcopenia assessment, nutritional status, activities of daily living, fall risk, and caregiver support	Low-start, slow-progression multimodal rehabilitation combining resistance, balance, functional task training, and aerobic conditioning	Falls risk, post-exertional exhaustion, adherence, nutrition/hydration, and caregiver-reported tolerance	^33–36,44)^
Mixed/triple-overlap phenotype	Combined ventilatory, hemodynamic, peripheral, and frailty-related limitations with high symptom variability	Multidomain assessment including CPET where feasible, echocardiography, renal function/electrolytes, spirometry, oxygenation, frailty, and nutritional status	Highly individualized, supervised, symptom-guided rehabilitation emphasizing realistic functional goals, conservative progression, and flexible FITT-VP adaptation	Volume status, oxygenation, blood pressure, symptoms, renal function, exacerbation/decompensation signals, and need for rapid escalation pathways	^4,5,7,51,66,68)^

CPET, cardiopulmonary exercise testing; peak VO_2_, peak oxygen uptake; VE/VCO_2_, minute ventilation/carbon dioxide production; FITT-VP, frequency, intensity, time, type, volume, and progression

Exercise prescription should follow the frequency, intensity, time, type, volume, and progression (FITT-VP) principle but be adapted according to dominant functional phenotypes—hemodynamic-limited, ventilatory-limited, peripheral-limited, or frailty-dominant—rather than applied uniformly based on diagnostic labels alone (Table 2). Aerobic training generally forms the foundation, often at moderate intensity, 3–5 days per week, with progression according to symptoms, exercise testing results, and clinical stability^8,9,52)^. Resistance training is particularly important for patients with sarcopenia, frailty, disability, or severe peripheral deconditioning and should be incorporated alongside aerobic training whenever feasible^9,44)^. Inspiratory muscle training can be a beneficial adjunct in selected patients with COPD or HF-COPD overlap who have prominent dyspnea or respiratory muscle weakness^56,62)^. Symptom-guided monitoring with Borg scales, heart rate, blood pressure, and oxygen saturation is essential, and exercise targets should remain flexible in patients whose day-to-day hemodynamic or respiratory status varies substantially.

Multiple delivery models are available. Center-based CR and PR provide direct supervision and ready access to multidisciplinary expertise and are typically preferred for high-risk patients, those with recent decompensation, and those with severe functional limitation^9,52,68)^. Home-based and hybrid programs may improve access and long-term adherence in selected lower-risk patients^54,69–71)^. Telerehabilitation should be conceptualized not merely as a substitute for center-based programs but as a structured delivery model that can provide core components of comprehensive rehabilitation when supported by appropriate patient selection, remote monitoring, and quality standards. In patients with multimorbidity, implementation should account for digital literacy, caregiver support, and the need for prompt escalation pathways when symptoms worsen.

Exercise-based rehabilitation in cardio-renal-pulmonary multimorbidity is generally safe when patients are clinically stable and programs are appropriately supervised. However, monitoring priorities differ according to phenotype. Volume status and renal function require particular attention in HF-CKD overlap, where rapid fluid shifts and diuretic adjustments may influence exercise tolerance^59,60)^. In COPD, exercise-induced desaturation and ventilatory limitation should be monitored closely, and supplemental oxygen should be considered when clinically indicated^52)^. In populations undergoing dialysis, intradialytic exercise is feasible when appropriately timed and monitored^41–43)^. Across all overlap phenotypes, safety depends not only on baseline disease severity but also on dynamic reassessment, individualized progression, and integration with broader chronic disease management (Table 2).

## Evidence Gaps and Future Directions

The evidence base for exercise-based rehabilitation in cardio-renal-pulmonary multimorbidity remains constrained by several important limitations. First, patients with advanced CKD, severe COPD, marked frailty, multiple comorbidities, or fluctuating clinical instability are frequently systematically excluded from major rehabilitation trials, limiting generalizability to the populations most in need of intervention^9,52)^. Second, dedicated RCTs in dual-overlap (HF-CKD and HF-COPD) and triple-overlap cardio-renal-pulmonary populations are scarce. Consequently, much of the available evidence is derived from subgroup analyses, observational studies, or small pilot trials^59,60,67)^. Third, major clinical outcomes—including mortality, hospitalization, COPD exacerbation, and validated renal endpoints—have not been sufficiently evaluated in multimorbid rehabilitation populations.

Future research should move beyond disease-specific trial design toward phenotype-adapted pragmatic studies that reflect real-world multimorbidity. Priority areas include: (1) adequately powered multicenter trials in overlap populations with clinically meaningful functional and patient-centered outcomes; (2) frailty-adapted interventions that combine aerobic and resistance training with nutritional and behavioral strategies; (3) better evaluation of renal endpoints and their measurement validity in exercise trials involving CKD; (4) investigation of biomarkers, CPET signatures, and imaging markers that predict response to rehabilitation; and (5) comparative studies of center-based, hybrid, home-based, and telerehabilitation models in high-risk multimorbid populations^50,66–68,70,71)^. A precision rehabilitation framework grounded in functional phenotype rather than diagnosis alone may be particularly valuable for the next generation of trials.

## Conclusions

Cardio-renal-pulmonary multimorbidity represents a growing clinical challenge characterized by complex pathophysiology, severe exercise intolerance, a high burden of frailty, and poor prognosis. The convergence of neurohormonal dysregulation, systemic inflammation, hemodynamic interdependence, endothelial dysfunction, and skeletal muscle impairment creates a self-reinforcing cycle of functional decline that pharmacological therapy alone cannot fully interrupt.

Exercise-based rehabilitation represents a biologically plausible, function-centered therapeutic strategy that targets shared pathophysiological mechanisms across cardiac, renal, and pulmonary systems, even in the absence of definitive evidence for hard clinical endpoints in complex multimorbidity. Evidence from single-disease populations supports improvements in exercise capacity, symptoms, and health-related QOL, and emerging data from overlap populations suggest that benefits remain achievable when programs are individualized, phenotype-guided, and carefully monitored. Clinicians managing these patients should move beyond disease-based classification alone and incorporate multimodal assessment, functional phenotyping, and personalized rehabilitation into routine care. Simultaneously, the research community must prioritize rigorous studies in multimorbid populations to define optimal intervention strategies, validate clinically meaningful endpoints, and establish a stronger evidence base to support high-quality rehabilitation in this underserved population.
